# Beyond Abscopal Effect: A Meta-Analysis of Immune Checkpoint Inhibitors and Radiotherapy in Advanced Non-Small Cell Lung Cancer

**DOI:** 10.3390/cancers13102352

**Published:** 2021-05-13

**Authors:** Francesco Fiorica, Umberto Tebano, Milena Gabbani, Mariasole Perrone, Sonia Missiroli, Massimiliano Berretta, Jacopo Giuliani, Andrea Bonetti, Andrea Remo, Eva Pigozzi, Andrea Tontini, Giuseppe Napoli, Nicoletta Luca, Daniela Grigolato, Paolo Pinton, Carlotta Giorgi

**Affiliations:** 1 Department of Radiation Oncology and Nuclear Medicine, AULSS 9 Scaligera, 37100 Verona, Italy; umberto.tebano@aulss9.veneto.it (U.T.); milena.gabbani@aulss9.veneto.it (M.G.); giuseppe.napoli@aulss9.veneto.it (G.N.); nicoletta.luca@aulss9.veneto.it (N.L.); daniela.grigolato@aulss9.veneto.it (D.G.); 2 Section of Experimental Medicine and Laboratory for Technologies of Advanced Therapies (LTTA), Department of Medical Sciences, University of Ferrara, 44123 Ferrara, Italy; prrmsl@unife.it (M.P.); sonia.missiroli@unife.it (S.M.); paolo.pinton@unife.it (P.P.); carlotta.giorgi@unife.it (C.G.); 3 Department of Clinical and Experimental Medicine, Policlinico “G. Martino” University of Messina, 98122 Messina, Italy; mberretta@unime.it; 4 Department of Medical Oncology, AULSS 9 Scaligera, 37100 Verona, Italy; jacopo.giuliani@aulss9.veneto.it (J.G.); andrea.bonetti@aulss9.veneto.it (A.B.); eva.pigozzi@edu.unife.it (E.P.); 5 Department of Pathology, AULSS 9 Scaligera, 37100 Verona, Italy; andrea.remo@aulss9.veneto.it; 6 Department of Mathematics and Computer Science, University of Ferrara, 44121 Ferrara, Italy; andrea.tontini@edu.unife.it

**Keywords:** combination of immune checkpoint inhibitors and radiotherapy, radiation oncology and immunity, radiation oncology, immunotherapy

## Abstract

**Simple Summary:**

Immune checkpoint inhibitors plus radiotherapy is emerging as a new strategy in non-small cell lung cancer patients. There were biological basis for this combination, the aim of this review of the literature was to explore clinical trials using this combination and to summarize the results of published studies with meta-analysis. The results of our systematic review should encourage the research community to further investigate the relationship between immune checkpoint inhibitors and radiotherapy, which may improve oncological outcomes.

**Abstract:**

Background: Immune checkpoint inhibitors (ICI) plus radiotherapy (RT) have been suggested as an emerging combination in non-small cell lung cancer (NSCLC) patients. However, little is known about the magnitude of its benefits and potential clinical predictors. Objective: To assess the effects of this combination on the increase in overall and progression-free survival. Data sources: The MEDLINE and CANCERLIT (1970–2020) electronic databases were searched, and the reference lists of included studies were manually searched. Study selection: Studies were included if they were comparative studies between combination ICI-RT and ICI or RT alone in advanced or metastatic NSCLC patients. Overall survival (OS) was analyzed according to the treatment strategy. Data extraction: Data on population, intervention, and outcomes were extracted from each study, in accordance with the intention-to-treat method, by two independent observers and combined using the DerSimonian method and Laird method. Results: Compared to ICI or RT alone, ICI-RT significantly increased the 1-year and 3-year OS RR by 0.75 (95% CI 0.64–0.88; *p* = 0.0003) and 0.85 (95% CI 0.78–0.93; *p* = 0.0006), respectively. Furthermore, there was a statistically significant benefit on 1- and 3-year progression-free survival (RR 0.73 (95% CI, 0.61–0.87; *p* = 0.0005) and RR 0.82 (95% CI 0.67–0.99; *p* = 0.04), respectively). Conclusions: In patients with advanced or metastatic NSCLC, combination ICI-RT increases 1- and 3-year OS and progression-free survival compared to ICI or RT alone.

## 1. Introduction

Tumor immunotherapy has been an amazing leap forward in cancer therapy. In particular, the introduction of immune checkpoint inhibitors into clinical practice has improved outcomes of lung cancer treatment [1]. This new family of drugs, anti-programmed cell death 1 (PD-1)/PD-L1, releases the inhibitory brakes to immunosuppression and consequently increases the immune response. The response to immunotherapy is conditioned by the pre-existing immune landscape within the tumor microenvironment [2]; therefore, immune checkpoint inhibitors only work when a lymphocyte infiltrate is present.

The interaction between radiation and the immune response has been experimentally proven. Radiation induces infiltration of a wide range of leukocytes: effector T cells, natural killer (NK) cells, regulatory T cells (Tregs), and CD11b-positive (CD11b+) cells, such as myeloid-derived suppressor cells (MDSCs) and tumor-associated macrophages (TAMs) [3]. As described by Kamvara et al. [4], radiation enhances the development of an antitumor immune response by inducing antigen release and immunogenic cell death, antigen presentation cell maturation and antigen presentation, T-cell recruitment and infiltration, and tumor cell sensitization to immune-mediated cell death. All these mechanisms serve as the basis for explaining the abscopal effect, a phenomenon described by Mole [5] as an action at a distance from the irradiated volume but within the same organism.

The efforts for immunomodulation to increase the effects of ICI-based treatment for advanced NSCLC favored the combination of ICI with RT in clinical practice [6] before it was demonstrated in RCTs. The combination of ICI-RT is under considerable debate and of clinical interest, as more advanced NSCLC patients are being treated with ICI-based treatment.

The results of published comparative studies remain inconsistent (mainly for small sample sizes), and an overall assessment of the treatment effect on survival is difficult to evaluate despite being clinically notable. The improvement in survival remains a major issue in the management of advanced or metastatic NSCLC. We aimed to summarize the available observational and randomized evidence in one updated systematic review and meta-analysis. Our objectives were to establish the effects of the ICI-RT association in terms of 1- and 3-year overall survival and 1-year and 3-year progression-free survival and to explore and explain the presence of heterogeneity among studies.

## 2. Methods

### 2.1. Selection of Studies

The search syntax of keywords was: “immune checkpoint inhibitor” OR “nivolumab” OR “pembrolizumab” OR “atezolizumab” OR “durvalumab” AND “Non-Small Cell Lung Cancer;” “immune checkpoint inhibitor” AND “Radiotherapy” AND “Lung Cancer;” “immune checkpoint inhibitor“ AND “Randomized Controlled Trial” OR “Comparative study.” Pertinent records were retrieved from MEDLINE, EMBASE, OVID, and Web of Science, and Scopus databases, while the timeframe applied was from the time of inception to 2020. No filters were applied regarding languages or countries to avoid language bias. To identify extra records, the computer search was supplemented with hand searches of reference lists for all available review articles, primary studies, meeting abstracts and bibliographies of books to identify additional studies not found in the computer search.

For grey literature and to avoid publication bias, Google Scholar were searched to retrieve unpublished relevant comparative studies. The total results from each online database and extra sources were recorded in a flow chart diagram of PRISMA. Finally, the number of results from each database was recorded. The final inclusion of articles was determined by consensus between 2 authors (F.F. and J.G.); discrepancies among reviewers were infrequent (overall interobserver variations <10%) and were solved by discussion.

All studies comparing survival outcomes between associations of ICI-RT and ICI or RT alone in patients with advanced NSCLC were deemed eligible for this meta-analysis. Because randomized controlled trials regarding this topic are rarely conducted, retrospective studies and nationwide population-based studies, such as the National Cancer Database (NCDB) study, were also included if they reported the survival outcomes.

The inclusion criteria were as follows: (1) the study enrolled a case group of patients treated with ICI and RT and a comparator group of patients treated with ICI alone; (2) the outcomes were compared in terms of OS; and (3) sufficient information was available to estimate the risk ratio (RR) and 95% confidence interval (CI). The eligible studies had to provide RR or crude data and corresponding standard errors (SE), variance, CI, or *p*-value of the significance of the estimates. Otherwise, the studies had to show the survival curves with the number in each group to estimate the RR.

The exclusion criteria were as follows: (1) studies analyzed fewer than 20 patients; (2) a single-arm design that did not have a control group; (3) case reports or only abstracts from conference meetings that were not published as original articles; and (4) more than 1 report by the same author or working group within the same study period.

### 2.2. Review of the Studies

Studies were first reviewed using a list of predefined, pertinent aspects concerning the characteristics of patients and treatments. The quality of each fully published trial was assessed. For observational cohort studies, we used the nine-star Newcastle-Ottawa Scale (NOS) [7], which is based on the predefined criteria of selection (population representativeness), comparability (adjustment for confounders), and ascertainment of the outcome. The NOS assigns a maximum of four points for selection, two points for comparability, and three points for the outcome. Nine points on the NOS reflect the highest study quality.

For RCTs, we used the Cochrane Collaboration’s risk of bias tool [8]. This tool assesses seven possible sources of bias: random sequence generation, allocation concealment, blinding of participants and personnel, blinding of outcome assessment, incomplete outcome data, selective reporting, and other bias. For each individual domain, studies were classified into low, unclear, and high risk of bias. Total methodologic quality scores were then used to rank the studies. Methodologic quality assessment was independently performed by two of the authors (U.T., G.N.). Discrepancies among reviewers were infrequent (overall interobserver variations of <10%) and were resolved by discussion.

### 2.3. Statistical Methods

The crude rates of 1-year and 3-year overall survival were assessed as measures of the treatment’s effect. If these data were not available [9], we used the Kaplan-Meier estimates of 1- and 3-year overall survival in the treated and control groups reported in the text. When possible, we also analyzed the 1-year and 3-year rates of progression-free survival. Evaluation of therapeutic effectiveness was performed with an intention-to-treat method. When not reported in the trial, the response rate according to intention-to-treat was calculated. Differences observed between the two groups were expressed as the pooled risk ratio (RR), with its 95% confidence interval (CI). The effect of treatment on the defined outcome measures was calculated from the study data using models based on both fixed and random effects assumptions. In addition to within-study variance, the random-effects model considers heterogeneity among studies. Because of the different clinical settings and groups of subjects analyzed and because the tests for heterogeneity lack statistical power due to the few studies included in this meta-analysis, we have presented the results of random-effects models introduced by DerSimonian and Laird [9]. Statistical heterogeneity between trials was evaluated using the Mantel-Haenszel *χ*^2^ test [10] and the I^2^ statistic [11].

The number needed to treat (NNT) for benefit, which derives from the inverse of the absolute difference in risk among treatment groups (1/RD), was also used as a measure of the benefits of treatment. We, in turn, excluded each study to ensure that no single study would be solely responsible for the significance of any result of the robust analysis. All these analyses were computed using Revman 5.3 (The Nordic Cochrane Centre, The Cochrane Collaboration, 2014. Copenhagen, Denmark).

We performed several sensitivity analyses to help address the heterogeneity of the study design and patient populations. Because National Cancer Database studies were present, we conducted separate analyses according to the source of data (RCTs vs. other studies) and study quality. We also analyzed studies according to comparators (RT alone vs. ICI alone) and baseline stage patients (locally advanced vs. metastatic). Finally, other study-level characteristics (such as PD-L1 status and histology) were prespecified for the assessment of heterogeneity, using subgroup analysis.

We assessed the potential for publication bias through formal tests (i.e., Egger’s regression intercept and classical fail-safe N) [12]. All statistical tests were two-sided and used a significance level of *p* < 0.05 using ProMeta software v.3.0 (Internovi, Cesena FC, Italy). Furthermore, we used the nonparametric approach reported by Combescure et al. [13] to assess the pooled overall survival over time. This approach returns a distribution-free summary survival curve by expanding the product-limit estimator of survival for aggregated survival data. R Core Team (2018): A language and environment for statistical computing (R Foundation for Statistical Computing, Vienna, Austria) were used to obtain this analysis and graphic. R code is obtainable at https://github.com/atontini/ICI-RT-NSCLC (accessed on 20 March 2021).

### 2.4. Role of the Funding Source

There was no funding source for this study. The corresponding author had full access to all the data in the study and had the final responsibility for the decision to submit it for publication.

## 3. Results

**Figure 1:**
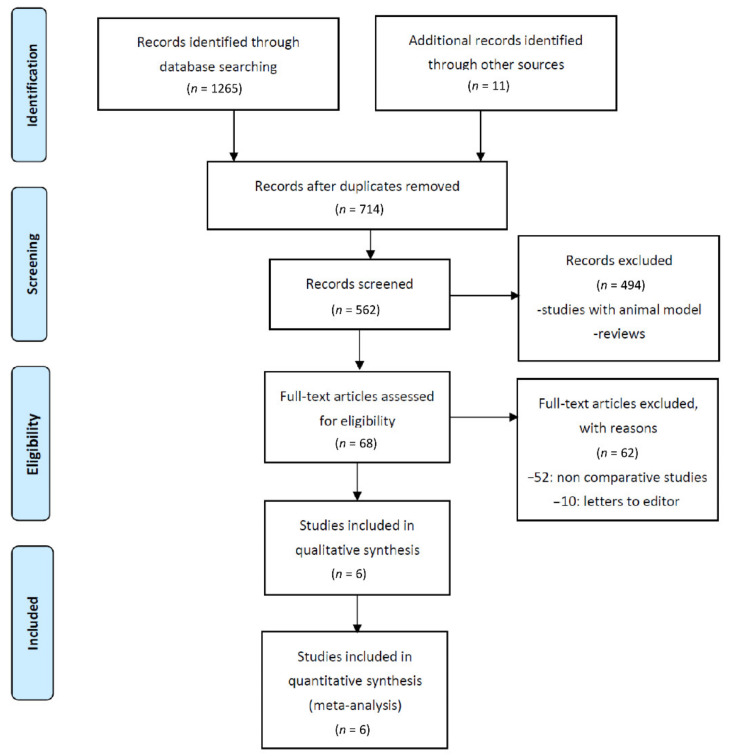
Flow chart of the literature search and selection process.

In total, 1276 records were initially retrieved from the computerized database search and manually checked. After the removal of duplicate records, 714 records were considered for review of the title and abstract. Of these, 82 records were selected as seemingly relevant publications. After assessment of the full text for eligibility, 68 records were further excluded because they were single-arm observation studies. Finally, six studies were included in the pooled analysis (Figure 1).

### 3.1. Characteristics of the Studies

The main features of the six trials included in this meta-analysis are shown in Table 1. These studies were published between 2016 and 2020 in three countries. Three trials were randomized [14,15,16], one was a subgroup analysis of a randomized trial [17], and two were National Cancer Database studies [18,19]. Together, the six studies [14,16,17,18,19] included 8435 patients, 4284 of who received only radiation therapy as a control arm and 4151 of whom received only immunotherapy. The main features of the trials included in the meta-analysis are shown in Table 1. The criteria to identify the study population were uniform: advanced NSCLC (810 patients) in two studies [14,15,17] and patients with metastatic NSCLC (7574 patients) in the remaining four studies [16,18,19]. The analyzed population of each study varied greatly, ranging from 76 17 to 3906 [18]. In two studies [14,15,19], ICI and RT associations were compared to RT alone and in the remaining four studies [16,17,18] to ICI alone. Data from two randomized trials [16,20] were extrapolated by a pooled analysis of the two studies [21]. The percentage of males ranged from 51.5% [17] to 95%. The mean patient age was 64.4 years, ranging from 62 [16] to 66.5. Data on the programmed-death-ligand 1 (PD-L1) status were shown in four studies [14,15,16,17], and PD-L1 <1% was present in 32.9% of patients, ranging from 27.6% [17] to 60.5% [14,15]. In all studies, radiotherapy was delivered before immunotherapy; in three studies [14,15,16], radiotherapy was planned within a protocol: using stereobody radiotherapy (SBRT) in two trials [16] and conventional radiotherapy in the Antonia et al. trial [14,15]. In the remaining three studies, radiotherapy was delivered previously either to their primary thoracic disease or to another metastatic site.

For the Bates et al. study [18], we chose to analyze patients only treated with hypofractionated radiotherapy (HRT). In this study, patients received HRT or standard fractionated radiotherapy (SFRT). In both settings, immunotherapy offered significantly improved OS. In those receiving HRT, immunotherapy improved the 1-year OS from 37.4% to 59.0%. In those receiving SFRT, immunotherapy improved the 1-year OS from 26.1% to 44.9%. The same methodology was applied to Foster et al.’s study [19]. In this study, there were two radiation groups: stereotactic radiotherapy and SFRT. The first group was associated with significantly improved OS, while the second group had significantly reduced OS.

### 3.2. Overall Survival

**Figure 2:**
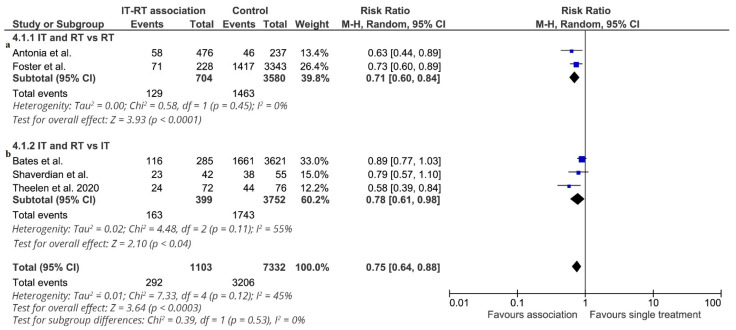
1-year overall survival. Meta-analysis of 5 studies of ICI-RT association: 2 studies of ICI-RT vs. RT alone (**a**) and 3 studies of ICI-RT vs. IT alone (**b**). The risk ratio (RR) and 95% confidence interval (CI) for the effect of treatment on 1-year overall survival are shown on a logarithmic scale. Studies are arranged by publication year. Theelen study is a pooled analysis of two RCTs.

The effect of adding radiotherapy to immunotherapy on 1-year overall survival (6 studies: 8435 patients, 1480 deaths) is shown in Figure 2. Data from two RCTs [16] were reported in the pooled analysis [21] of the two randomized clinical trials. The effect of treatment on 1-year overall survival significantly favored the association of ICI-RT in all studies, and a statistically significant difference was observed in three studies. The RR of each trial ranged from 0.63 to 0.89. The pooled estimate of the treatment effect was significant (RR 0.75, (95% CI 0.64–0.88); *p* = 0.0003) (NNT = 11). Similar results were obtained when a fixed-effects model was used (RR 0.78, (95% CI 0.70–0.86); *p* < 0.00001). Using robust analysis, the pooled estimate of the treatment effect was significant. In particular, robust analyses showed that evaluation of the five studies remaining after omission of the largest trial by Bates et al. [18] did not lose statistical significance for 1-year overall survival (RR 0.70 (95% CI 0.61–0.81)). In the subgroup of studies comparing ICI-RT vs. RT alone (4284 patients), the association of ICI-RT was associated with a statistically significant benefit in 1-year OS (RR 0.71, (95% CI 0.60–0.84) *p* < 0.0001) (NNT = 11). In the subgroup of studies comparing ICI-RT vs. ICI alone (4151 patients), the addition of RT to immunotherapy was associated with a statistically significant benefit in 1-year OS (RR 0.78, (95% CI 0.61–0.98) *p* = 0.04) (NNT = 7). Analyzing two studies [14,17] with locally advanced NSCLC, treatment with ICI-RT was associated with a statistically significant benefit in 1-year OS (RR 0.71, (95% CI 0.56–0.91) *p* = 0.006) (NNT = 12). In the subgroup of studies enrolling metastatic NSCLC, ICI-RT was associated with a statistically significant 1-year OS benefit (RR 0.76, (95% CI 0.61–0.94) *p* = 0.02) (NNT = 9).

**Figure 3:**
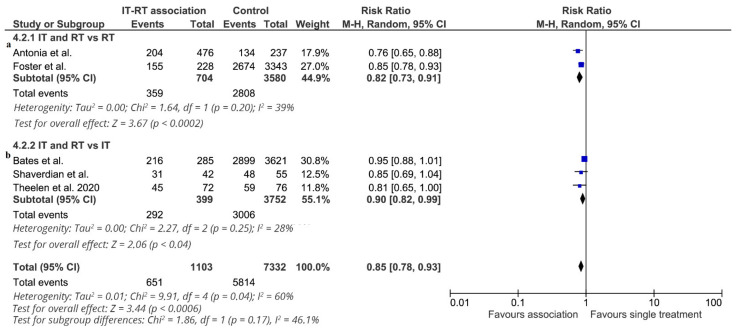
3-year overall survival. Meta-analysis of 5 studies of ICI-RT association: 2 studies of ICI-RT vs. RT alone (**a**) and 3 studies of ICI-RT vs. ICI alone (**b**). The risk ratio (RR) and 95% confidence interval (CI) for the effect of treatment on 3-year overall survival are shown on a logarithmic scale. Studies are arranged by publication year. Theelen study is a pooled analysis of two RCTs.

Three-year overall survival was reported in six studies (8435 patients: 3298 deaths). Data from two RCTs [16] were reported in the pooled analysis [21] of these two randomized clinical trials. The effect of the association of ICI-RT on 3-year overall survival is shown in Figure 3. The association of ICI-RT increased 3-year OS in all studies, and a significant difference was observed in two studies [14,19]. The RR of each study ranged from 0.78 to 0.95. The pooled estimate of the treatment effect was significant (RR 0.85, (95% CI 0.78–0.93) *p* = 0.0006) (NNT = 10). Similar results were obtained when a fixed effect model used RR 0.61 (95% CI 0.52–0.72; *p* < 0.00001). This effect on 3-year overall survival depended on the study. In the subgroup of studies comparing ICI-RT vs. RT alone (4284 patients), treatment with ICI-RT was associated with a statistically significant benefit in 3-year OS (RR 0.82, (95% CI 0.73–0.91) *p* < 0.00001) (NNT = 7). In the subgroup of studies comparing ICI-RT vs. ICI alone (4284 patients), ICI-RT was associated with a marginally statistically significant benefit in 3-year OS (RR 0.90, (95% CI 0.82–0.99) *p* = 0.04) (NNT = 12). Analyzing two studies [14,17] with locally advanced NSCLC, treatment with ICI-RT was associated with a statistically significant benefit in 3-year OS (RR 0.79, (95% CI 0.70–0.89) *p* = 0.0001) (NNT = 7). In the subgroup of studies enrolling metastatic NSCLC, treatment with ICI-RT was associated with a significant benefit in 3-year OS (RR 0.89, 95% (CI 0.81–0.97) *p* = 0.01) (NNT = 11).

**Figure 4:**
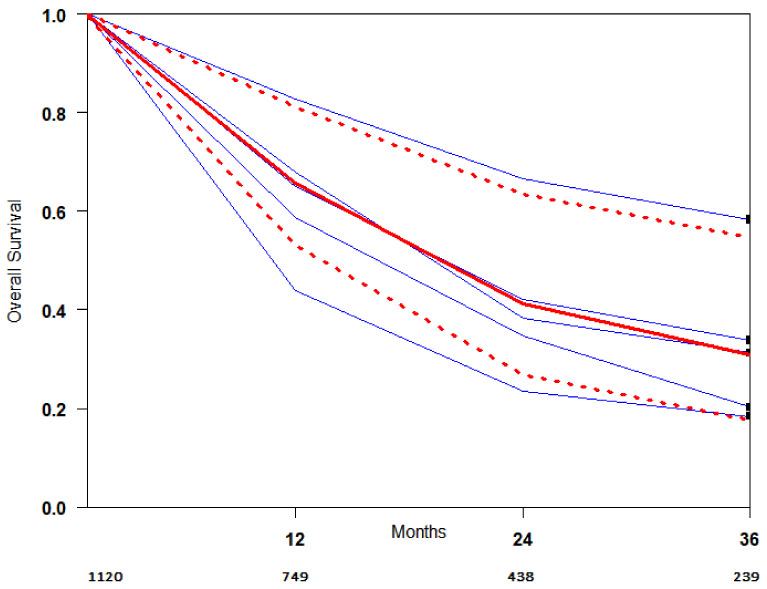
OS curve of studies included in the meta-analysis. Blue lines represent survival curves for each study. The red thick line represents the summarized survival curve with the 95% confidence bands (dashed lines) obtained using the approach of Combescure et al. with random effects.

OS curves for the ICI-RT association were extracted from the studies, and the summary survival curves are shown in Figure 4. The median survival (95% CI) was 19.7 months.

### 3.3. 1-Year and 3-Year Progression-Free Survival

**Figure 5:**
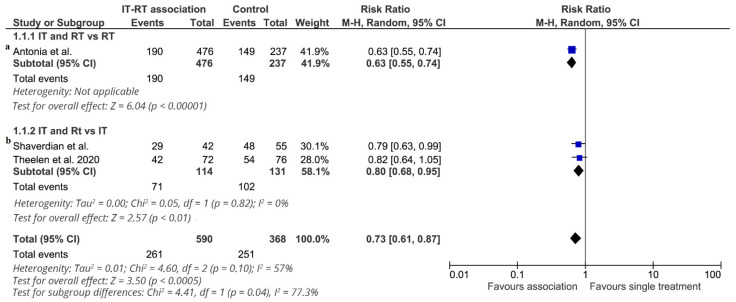
1-year progression-free survival. Meta-analysis of 3 studies of ICI-RT association: 1 study of ICI-RT vs. RT alone (**a**) and 2 studies of ICI-RT vs. ICI alone (**b**). The risk ratio (RR) and 95% confidence interval (CI) for the effect of treatment on 1-year progression-free survival are shown on a logarithmic scale. Studies are arranged by publication year. Theelen study is a pooled analysis of two RCTs.

The effect of ICI-RT on 1-year progression-free survival (4 studies: 958 patients, 512 subjects without disease progression) is shown in Figure 5. The pooled estimate of the treatment effect on 1-year progression-free survival was significant (RR 0.73 (95% CI, 0.61–0.87) *p* = 0.0003) (NNT = 4). Similar results were obtained when a fixed-effects model was used. This effect differed across studies. Only one study compared ICI-RT vs. RT alone [14,15] (713 patients). ICI-RT was associated with a statistically significant benefit (RR 0.63, (95% CI 0.55–0.74) *p* < 0.000001) (NNT = 4). In the subgroup of studies comparing ICI-RT vs. ICI alone (245 patients), the association of RT with immunotherapy was associated with a statistically significant benefit in 1-year PFS (RR 0.80, (95% CI 0.68–0.95) *p* = 0.01). Two studies [14,15,17] enrolled locally advanced NSCLC patients, and treatment with ICI-RT was associated with a statistically significant benefit in 1-year PFS (RR 0.70, (95% CI 0.56–0.87) *p* = 0.001) (NNT = 4). Two studies enrolled metastatic NSCLC [16], and data were extrapolated by a pooled analysis of the two RCTs [21]. ICI-RT was not associated with a statistically significant 1-year PFS benefit (RR 0.82, (95% CI 0.64–1.05) *p* = 0.11).

**Figure 6:**
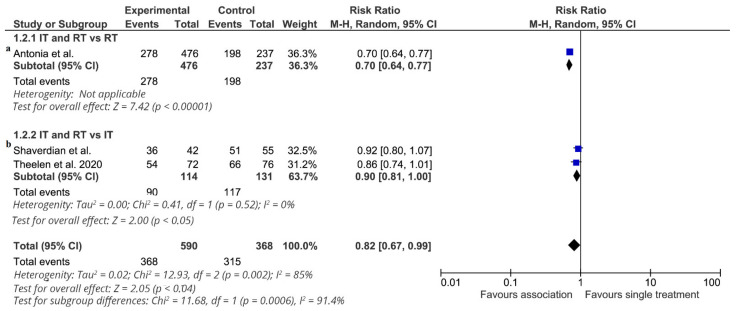
3-year progression-free survival. Meta-analysis of 3 studies of ICI-RT association: 1 study of ICI-RT vs. RT alone (**a**) and 2 studies of ICI-RT vs. ICI alone (**b**). The risk ratio (RR) and 95% confidence interval (CI) for the effect of treatment on 3-year progression-free survival are shown on a logarithmic scale. Studies are arranged by publication year. Theelen study is a pooled analysis of two RCTs.

Furthermore, the effect of additional radiotherapy on 3-year progression-free survival (4 studies: 958 patients, 255 subjects without disease progression) is shown in Figure 6. The effect of treatment on 3-year progression-free survival favored the association in all studies, and a statistically significant difference was observed in only one study [14,15]. The RR of each trial ranged from 0.70 to 0.92. The pooled estimate of the treatment effect was significant (RR 0.82 (95% CI 0.67–0.99) *p* = 0.04) (NNT = 6). Similar results were obtained when a fixed-effects model was used (RR 0.75 (95% CI 0.70–0.81) *p* < 0.00001). In all the robust analyses, the pooled estimate of the treatment effect was significant.

Only one study compared ICI-RT vs. RT alone [14,15] (713 patients). ICI-RT was associated with a statistically significant benefit (RR 0.70 (95% CI 0.64–0.77) *p* < 0.000001) (NNT = 4). In the subgroup of studies comparing ICI-RT vs. ICI alone (245 patients), the association of RT with immunotherapy was associated with a statistically significant benefit in 3-year PFS (RR 0.89 (95% CI 0.80–0.99) *p* = 0.04) (NNT = 10). Two studies [14,15,17] enrolled locally advanced NSCLC patients, and ICI-RT did not significantly increase 3-year PFS (RR 0.80 (95% CI 0.60–1.06) *p* = 0.13). Two studies enrolled metastatic NSCLC [16], and data were extrapolated by a pooled analysis of the two RCTs [21]. ICI-RT was not associated with a statistically significant benefit in 3-year PFS (RR 0.86 (95% CI 0.74–1.01) *p* = 0.07).

### 3.4. Subgroup Analysis

The results of the sensitivity analysis for 1- and 3-year overall survival and progression-free survival are shown in Table 2. Separate subgroup analyses were performed in relation to the type of study (RCTS vs. other studies), quality score (excluding trials of low quality), comparators (RT vs. ICI alone), population (locally advanced NSCLC vs. metastatic NSCLC), rate of PD-L1 < 1% (<20% vs. >20%) and rate of adenocarcinoma (<75% vs. >75%. All tests for homogeneity showed no statistical significance, leaving the overall effect and confidence intervals essentially unchanged. Furthermore, the difference between 1- and 3- year overall survival and progression-free survival was comparable by analyzing studies with a rate > 20% of PD-L1 < 1% and with a rate > 75% of adenocarcinoma.

### 3.5. Safety

**Figure 7:**
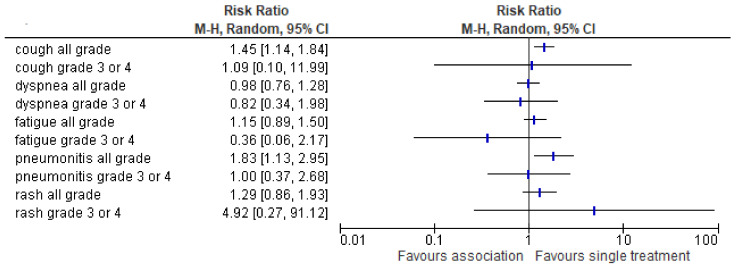
The risk ratio of the most frequent adverse events in the association treatment group as compared to the single Table 95. confidence interval (CI) for the adverse effect rate are shown on a logarithmic scale. Adverse events are arranged by alphabetic order (all grade AE and 3–4 grade, respectively).

Safety is reported in 3 RCTs analyzing 821 patients. Maximum-grade 3 or 4 adverse events of any cause occurred in 31.3% of the patients in the ICI-RT group and in 25.9 % of those in the single treatment (26.1% in radiotherapy alone group and 25% in ICI alone). Discontinuation of the study treatment because of adverse events occurred in 14.1% of the patients in the associated treatment group and in 8.6% of those in the single treatment. No statistically significant differences were found between the most frequent adverse events (Figure 7).

### 3.6. Publication Bias

Based on Begg’s test and Egger’s test for small-study effects, there was no publication bias. In Egger’s test, the *p*-values were 0.07 for 1-year OS and 0.13 for 3-year OS and 0.86 and 0.23 for 1- and 3-year PFS, respectively.

According to the classical fail-safe N approach, 30 negative studies are required to nullify the effect of the association on 1-year overall survival and 32 on 3-year overall survival. Twenty-one studies are required to nullify the effect of the association on 1-year PFS, and 25 are required for 3-year PFS.

## 4. Discussion

Immunotherapy has been explored extensively in clinical trials as monotherapy for different solid cancers. For immune checkpoint inhibitors, combinatory use is still under investigation. A recent meta-analysis suggests that overall survival and progression-free survival in NSCLC can be improved by combination with other drugs, such as paclitaxel and carboplatin, dacarbazine, sargramostim, and the Gp100 vaccine [22]. Another recent systematic review summarized drugs, molecules, and viruses that could improve the efficacy of ICIs, evaluating immunological mechanisms, which lead to enhance this anti-cancer efficacy [23].

Hence, this study investigated the key clinical question of whether adding RT to ICI could be more efficacious than ICI or RT alone in locally advanced or metastatic NSCLC patients.

In this meta-analysis of literature data from 6 comparative studies analyzing 8435 patients, we found that ICI-RT improved 1- and 3-year overall survival versus ICI or RT alone. Furthermore, a pooled actuarial curve of OS was obtained (Figure 4) using Combescure et al. [13] method. A summary survival curve of ICI-RT patients meets a real need in clinical research to use as a reference for determining the sample size of future systemic studies comparing new associations and for obtaining indirect comparisons among different trials estimating treatment efficacy.

RT with concurrent chemotherapy (CT) is the standard of care for the majority of patients with stage III NSCLC with radical intent. Additionally, in stage III NSCLC patients not eligible for radical treatment, an advantage in the survival of RT and CT has clearly been demonstrated [24,25]. In stage IV NSCLC, RT is considered only for palliative intent; however, there is a group of metastatic patients in whom ablative RT to metastatic sites may result in long-term survival [26]. To date, this is the standard approach, and the introduction of molecules unleashing the immune response against cancer in clinical practice can modify the treatment approach. RT has the ability to convert irradiated tumors into immunogenic hubs [27], and available in vitro and in vivo data have shown the ability of radiation to synergize with immunotherapy. This ability is the basis for the so-called abscopal effect; however, it is unclear how and when this effect takes place. The abscopal effect, the interaction of radiation with no target, is a fairly rare effect, and a systematic review collecting abscopal cases [28] described only 46 patients from 1969 to 2014. A few years ago, a proof of principle trial showed that the combination of radiotherapy with granulocyte-macrophage colony-stimulating factor elicited an abscopal response in 11 patients with solid metastatic tumors, increasing overall survival from a median of 8.3 to 20.9 months [29]. This randomized trial led to an interaction between host immune status and radiotherapy from bench to bedside. To answer the question of why abscopal effects of RT are so rare, Dovedi et al. [30] showed that PDL1, an immunosuppressive ligand, is upregulated in tumor cells after irradiation. Hence, one can speculate that this upregulation results in a braking effect of immunological cells carrying in tumors after irradiation. Therefore, a barrier is established for cellular immunity against tumor cells. Immune checkpoint inhibitors that block the interaction of PD-L1 with the PD-1 receptor can strengthen synergism with radiotherapy. A recent pooled analysis of two RCTs using pembrolizumab [21] showed an abscopal response rate increase of 22% as an “absolute increase” and 111% as a “relative increase,” which resulted in a surprisingly low NNT to obtain an abscopal effect in 4.5 patients. This abscopal rate could be considered a surrogate response to progression-free survival and overall survival and could open the door for new treatment paradigms. Our meta-analysis has the strength to include observational evidence and clinical trials in a single study, thus providing the most comprehensive update on the effect of the association of RT and immunotherapy. We did not evaluate the abscopal rate but evaluated the 1- and 3-year PFS and 1- and 3-year overall survival rates. ICI-RT associations globally decreased the risk of death at 1 year by 24% in patients and at 3 years by 15% in patients. A similar reduction in the death rate was shown by a meta-analysis of only RCTs comparing the association of ICI with CT vs. ICI alone in advanced NSCLC [31]. Interestingly, a statistically significant benefit was maintained by pooling RCTs (reduction of risk of death at 1 and 3 years by 48% and 26%, respectively) and pooling the two real-life studies [18,19] (reduction of risk of death at 1 and 3 years by 18% and 12.5%, respectively). Because our meta-analysis was based on both observational and clinical trial evidence, our findings underscore a potential beneficial role of ICI-RT. Our findings also confirmed that ICI-RT had a PFS advantage over ICI or RT monotherapy, with a reduction in the risk of progression at 1 and 3 years by 35% and 13%, respectively.

Considering the data available, we were able to systematically explore possible sources of heterogeneity using stratified analyses. Pooling studies with more than 20% of PD-L1 <1%, there is no change in the difference of outcomes. These findings are consistent with the results of two published studies [14,15,16], suggesting that the PD-L1–negative subgroup showed a significant benefit from the combined approach. Normally, response rates of ICI-treated patients with NSCLC are supposed to be dependent on the PD-L1 expression levels of the tumor [32,33]. Because RT was delivered before immunotherapy in all studies, it is likely that RT is able to increase the PL-D1 rate in irradiated tumor cells, bypassing problems related to PD-L1 expression that are intrinsic to ICI therapy [34]. These clinical data reinforce biological data [30], clearly demonstrating that irradiation of tumor cells upregulates PD-L1 ligands. Further studies are needed to understand more fully.

Pooling studies with a rate of > 75%, the difference in 1- and 3- year overall survival and progression-free survival was comparable.

The results of this retrospective analysis are subject to several limitations. Differences in the baseline severity of illness in the population of the studies, the irradiation dose and fractionation and the drug used may have limited the accuracy of this meta-analysis. These summary results describe variation only between studies, not between patients, because they reflect group averages rather than individual data. Lack of data on other potential confounders could also have affected the accuracy of the results. The meta-analysis was performed using summary data, and more detailed treatment comparisons could be achieved with a meta-analysis of individual patient data. Screening of the non-English literature and extensive manual and computer searches for studies made us confident that no important published trials were overlooked. A detailed assessment of the methodological quality of the included studies was performed; observational cohort studies were all of adequate quality. The study should ideally have been registered in a database such as PROSPERO before the study start to avoid research duplication, promotes transparency and help reduce potential for bias.

Publication bias was probably not substantial and considered unlikely to change the direction of our pooled estimate of the treatment effect. We should be particularly concerned about publication bias in settings in which small studies are being conducted.

## 5. Conclusions

The evidence from the literature data is sufficient to conclude the following: (1) the addition of immunotherapy to radiotherapy or immunotherapy alone improves 1- and 3-year overall survival; (2) progression-free survival is significantly influenced by ICI-RT association; and (3) gain in overall survival seems be retained in adenocarcinoma and PD-L1 negative patients.

## Figures and Tables

**Table 1:** Characteristics of NSCLC patients in studies included in the meta-analysis.

Trial	Comparison	Total *N* Patients	Male	Age	Histology	Setting	PD-L1 < 1%	PD-L1 > 1%	Objective Response Rate	Comparative Regimens	Assessed Study Quality
			(%)								
Phase III RCT											
Antonia et al. [14,15]	ICI-RT vs. RT alone	237	70.1	64	Adeno: 387 Squamous: 326	Advanced	148	303	365	RT	Low risk of bias ****
		476								RT + DURVA	
Theelen et al. [16]	ICI-RT vs. ICI alone	40	56.6	<65:43	Adeno: 67 Squamous: 9	Metastatic	43	31	26	PEMBRO	Low risk of bias ****
		36		≥65:33						PEMBRO + RT	
Welsh el al. [20]	ICI-RT vs. ICI alone	40	63.7	66	Adeno: 61 Squamous: 17	Metastatic	19	32	17	PEMBRO	Low risk of bias ****
		40								PEMBRO + RT	
NCDB											
Foster et al. [19] *	ICI-RT vs. RT alone	3344	52.2	64.5	Adeno: 2825 Squamous: 746	Metastatic	n.r.	n.r.	n.r.	RT	7/9 ***
		228								RT + ICI	
Bates el al. [18] **	ICI-RT vs. ICI alone	3621	53.2	<65:3165	Adeno: 3472 Squamous: 434	Metastatic	n.r.	n.r.	n.r.	ICI	7/9 ***
		285		≥65:3218						RT + ICI	
Subgroup analysis of RCT											
Shaverdian et al. [17]	ICI-RT vs. ICI alone	42	51.5	65.6	Adeno: 78 Squamous: 19	Advanced	11	55	19	PEMBRO	Unclear risk of bias ****
		55								RT + PEMBRO	

Legend: *N* = number; NCDB = National Cancer Database; NSCLC = non-small cell lung cancer; ICI = immunotherapy; DURVA = durvalumab; PEMBRO = pembrolizumab; n.r. = not reported; RT= radiotherapy; RCT = randomized controlled trial. * data related to patients treated with immunotherapy with no radiotherapy or with stereobody radiotherapy; ** data related to patients treated with immunotherapy with no radiotherapy or with stereobody radiotherapy; *** Quality assessed with nine-star Newcastle-Ottawa Scale (NOS); **** Quality assessed with Cochrane Collaboration’s risk of bias tool.

**Table 2:** Sensitivity analysis.

Strata of Sensitivity-Analysis Results for Each End Point	Subgroup (*n*)	References	RR (95% CI)	*p*-Value
**Exclusion of NCDB studies**				
1-year OS	Only RCTs (958)	[15,17,18]	0.60 [0.47, 0.78]	0.0001
3-year OS	Only RCTs (958)	[15,17,18]	0.79 [0.71, 0.88]	<0.0001
**Exclusion of low-quality studies**				
1-year OS	Only high quality (8338)	[16,18,19,17,22]	0.74 [0.61, 0.89]	0.002
3-year OS	Only high quality (8338)	[16,18,19,17,22]	0.85 [0.77, 0.95]	0.003
1-year PFS	Only high quality (861)	[15,17,18]	0.71 [0.55, 0.91]	0.007
3-year PFS	Only high quality (861)	[15,17,18]	0.77 [0.63, 0.95]	0.01
**Exclusion of studies without metastatic disease**				
1-year OS	Only metastatic patients (7625)	[16,17,18,20]	0.76 [0.61, 0.94]	0.01
3-year OS	Only metastatic patients (7625)	[16,17,18,20]	0.89 [0.81, 0.97]	0.01
1-year PFS	Only metastatic patients (148)	[17,18]	0.82 [0.64, 1.05]	0.11
3-year PFS	Only metastatic patients (148)	[17,18]	0.86 [0.74, 1.01]	0.07
**Exclusion of studies with RT alone as control arm**				
1-year OS	Only patients treated with ICI as control arm (4151)	[18,19,17,20,22]	0.78 [0.61, 0.98]	0.04
3-year OS	Only patients treated with ICI as control arm (4151)	[18,19,17,20,22]	0.90 [0.82, 0.99]	0.04
1-year PFS	Only patients treated with ICI as control arm (245)	[17,18]	0.80 [0.68, 0.95]	0.01
3-year PFS	Only patients treated with ICI as control arm (245)	[17,18]	0.90 [0.81, 1.00]	0.05
**Exclusion of studies with < 20 % rate of PD-L1 negative**				
1-year OS	Only studies with a rate > 20% of PD-L1 negative (869)	[14,15,16,20]	0.60 [0.47, 0.78]	0.0001
3-year OS	Only studies with a rate > 20% of PD-L1 negative (869)	[14,15,16,20]	0.77 [0.68, 0.88]	0.0001
1-year PFS	Only studies with a rate > 20% of PD-L1 negative (869)	[14,15,16,20]	0.71 [0.55, 0.91]	0.0007
3-year PFS	Only studies with a rate > 20% of PD-L1 negative (869)	[14,15,16,20]	0.77 [0.63, 0.95]	0.01
**Exclusion of studies with < 75 % rate of adenocarcinoma**				
1-year OS	Only studies with a rate > 75% of adenocarcinoma (7731)	[16,17,18,20]	0.60 [0.47, 0.78]	0.0001
3-year OS	Only studies with a rate > 75% of adenocarcinoma (7731)	[16,17,18,20]	0.88 [0.82, 0.96]	0.002
1-year PFS	Only studies with a rate > 75% of adenocarcinoma (253)	[16,22]	0.80 [0.68, 0.95]	0.01
3-year PFS	Only studies with a rate > 75% of adenocarcinoma (256)	[16,22]	0.90 [0.81, 1]	0.05

## Data Availability

The datasets analyzed during the current study are available from the corresponding author on reasonable request.

## Abbreviations

CI	confidence interval
CT	chemotherapy
DURVA	durvalumab
ICI	immune checkpoint inhibitors
HRT	hypofractionated radiotherapy
MDSCs	myeloid-derived suppressor cells
NCDB	National Cancer Database
NK	natural killer
NNT	number needed to treat
NOS	Newcastle-Ottawa Scale
NSCLC	non-small cell lung cancer
OS	overall survival
PEMBRO	pembrolizumab
PD-1	programmed cell death
RR	risk ratio
RT	radiotherapy
SBRT	stereobody radiotherapy
SE	standard errors
SFRT	standard fractionated radiotherapy
TAMs	tumor-associated macrophages
Treg	regulatory T cells
